# Gap junctions contain different amounts of cholesterol which undergo unique sequestering processes during fiber cell differentiation in the embryonic chicken lens

**Published:** 2007-03-09

**Authors:** Sondip K. Biswas, Woo-Kuen Lo

**Affiliations:** 1 Depatment of Anatomy & Neurobiology, Morehouse School of Medicine, Atlanta, GA; 2 Department of Ophthalmology, Emory University, Atlanta, GA

## Abstract

**Purpose:**

To determine the possible changes in the distribution of cholesterol in gap junction plaques during fiber cell differentiation and maturation in the embryonic chicken lens. The possible mechanism by which cholesterol is removed from gap junction plaques is also investigated.

**Methods:**

Filipin cytochemistry in conjunction with freeze-fracture TEM was used to visualize cholesterol, as represented by filipin-cholesterol complexes (FCCs) in gap junction plaques. Quantitative analysis on the heterogeneous distribution of cholesterol in gap junction plaques was conducted from outer and inner cortical regions. A novel technique combining filipin cytochemistry with freeze-fracture replica immunogold labeling (FRIL) was used to label Cx45.6 and Cx56 antibodies in cholesterol-containing gap junctions. Filipin cytochemistry and freeze-fracture TEM and thin-section TEM were used to examine the appearance and nature of the cholesterol-containing vesicular structures associated with gap junction plaques.

**Results:**

Chicken lens fibers contain cholesterol-rich, cholesterol-intermediate and cholesterol-free gap junction populations in both outer and inner cortical regions. Filipin cytochemistry and FRIL studies confirmed that cholesterol-containing junctions were gap junctions. Quantitative analysis showed that approximately 86% of gap junctions in the outer cortical zone were cholesterol-rich gap junctions, whereas approximately 81% of gap junctions in the inner cortical zone were cholesterol-free gap junctions. A number of pleiomorphic cholesterol-rich vesicles of varying sizes were often observed in the gap junction plaques. They appear to be involved in the removal of cholesterol from gap junction plaques through endocytosis.

**Conclusions:**

Gap junctions in the young fibers are enriched with cholesterol because they are assembled in the unique cholesterol-rich cell membranes in the lens. A majority of cholesterol-rich gap junctions in the outer young fibers are transformed into cholesterol-free ones in the inner mature fibers during fiber cell maturation. A distinct endocytotic process appears to be involved in removing cholesterol from the cholesterol-containing gap junctions, and it may play a major role in the transformation of cholesterol-rich gap junctions into cholesterol-free ones during fiber cell maturation.

## Introduction

Lens fiber cell membrane contains the richest cholesterol content in all cell type membranes in the body [1-7]. The cholesterol to phospholipids (C/P) molar ratio ranges from 1 to 4 from the lens cortex to lens nucleus, while that of typical eukaryotic cells is between 0.5 and 1.0 [1,2,7,8]. Since all gap junctions are assembled in the cholesterol-rich cell membranes, it is reasonable to expect that many newly-formed gap junctions will be enriched with cholesterol in the cortical fiber cells. Several early biochemical studies have indeed shown that isolated fiber gap junction fractions from several species, as verified by electron microscopy, contain rich cholesterol and other lipid components [9-11]. Since the lens fiber gap junctions do not turnover as rapidly as those gap junctions in other tissues [12-18], the newly formed cholesterol-rich gap junctions in the superficial fiber cells would undergo the maturation process during fiber differentiation and maturation.

The specific distribution of cholesterol in fiber gap junctions has not yet been studied morphologically in details. We are interested to find out whether the distribution and content of cholesterol in gap junctions change during fiber cell differentiation and maturation. We used the embryonic chicken as our choice of an animal model because embryonic chicken lenses at various ages posses a large number of gap junctions [19-22].

In this study, by using filipin cytochemistry in conjunction with freeze-fracture TEM for high resolution examinations of cholesterol distribution in the cell membrane [23-25], we are able to conduct systematic structural and quantitative analyses on the distribution of cholesterol in gap junction plaques from superficial to deep cortical fiber cells. We have found that fiber gap junctions contain heterogeneous amounts of cholesterol in different cortical regions. There are cholesterol-rich as well as cholesterol-poor and -free gap junctions distributed in both outer and inner cortical fiber cells in the embryonic chicken lens. Quantitative analysis showed that approximately 86% of gap junctions belong to "cholesterol-rich" groups in the outer cortex. In contrast, approximately 81% of gap junctions belong to "cholesterol-poor" and "cholesterol-free" groups in the inner cortex. These results suggest that a majority of cholesterol-rich gap junctions in the young outer cortical fibers have transformed into cholesterol-poor and -free gap junctions in the mature inner cortical fibers during fiber cell maturation. Furthermore, we have observed a new endocytotic process for internalization of cholesterol-rich vesicles which are specifically associated with gap junction plaques in both outer and inner cortices during fiber differentiation and maturation. We hypothesize that endocytosis of cholesterol-rich vesicles is one of the sequestering processes for removing cholesterol from the cholesterol-rich gap junctions to become the cholesterol-poor and -free ones during gap junction maturation and aging.

## Methods

### Collection of chicken embryonic lenses

Fertile white leghorn chicken eggs (Hyline International, Mansfield, GA) were incubated at 38 °C in a humidified incubator (Petersime, Gettysburg, OH) for 4-20 days. After the appropriate incubation period, the embryonic lenses were surgically isolated. The animals were treated in accordance with the Association for Research in Vision and Ophthalmology Resolution on the Use of Animals in Research.

### Systematic detection of cholesterol in cell membranes by specific filipin-cholesterol complexes (FCC) with freeze-fracture TEM

Chicken embryonic lenses from E12-E20 were enucleated, cleaned in 0.1 M cacodylate buffer, and fixed in 2.5% glutaraldehyde in 0.1 M cacodylate buffer (pH 7.3) at RT for 2 h. After washing in buffer, lenses were mounted on specimen holders with superglue and agar and cut into 300 μm slices with a Vibratome. The lens was orientated initially (i.e., mounting the lens equator up) to obtain sagittal (longitudinal) sections with Vibratome and the sections were collected and marked serially from superficial to deep and kept separately. The slices were then incubated in a mixture of 2.5% glutaraldehyde in 0.1 M cacodylate buffer and 0.1% filipin in dimethyl formamide (Sigma, St. Louis, MO) for 24 h. These slices were then processed for freeze-fracture TEM according to our routine procedures [26] to visualize the filipin-cholesterol complexes (FCCs). In brief, the tissue slices were cryoprotected with 25% glycerol in 0.1 M cacodylate buffer at RT for 1 h. Each slice was mounted on a gold specimen carrier and frozen rapidly in the liquefied Freon 22 and stored in liquid nitrogen. Cryofractures of frozen slices were made in a modified Balzers 400T freeze-fracture unit, at a stage temperature of -135 °C in a vacuum of approximately 2x10-7 Torr. The lens tissue was fractured by scraping a steel knife across a frozen surface to explore fiber cell membranes. The fractured surface was immediately replicated with platinum (about 2 nm thick) followed by carbon film (about 25 nm thick). The replicas, obtained by unidirectional shadowing, were cleaned with household bleach and examined with the electron microscope. The FCCs are discrete particles or pits (25-35 nm in diameter) which can be clearly visualized on the P face and E face of the plasma membrane with freeze-fracture TEM. The formation of FCCs is due to the polyene antibiotic filipin reacting specifically with membrane cholesterol which produces characteristic membrane lesions seen as the FCCs. The filipin cytochemistry and freeze-fracture procedures were according to established procedures [23-25] with modifications. In order to determine that we have provided enough incubation time for filipin to diffuse into the entire lens slices to react with membrane cholesterol, we have tested a number of incubation times, ranging from 8-48 h. We have obtained all similar results. The distinct filipin-cholesterol complexes can also be consistently obtained by incubating the lens slices with 0.1% filipin solution alone after a brief fixation with 0.75-1% paraformaldehyde-PBS for 30-45 min at RT. With these lightly-fixed samples, we have successfully developed a novel technique combining filipin cytochemistry and freeze-fracture replica immunogold labeling (FRIL) for simultaneous localization of both cholesterol and antibodies of interest in fiber cell membranes in this study.

### High resolution freeze-fracture replica immunogold labeling (FRIL) of Cx45.6 and Cx56 in lens fiber cells

**Figure 1:**
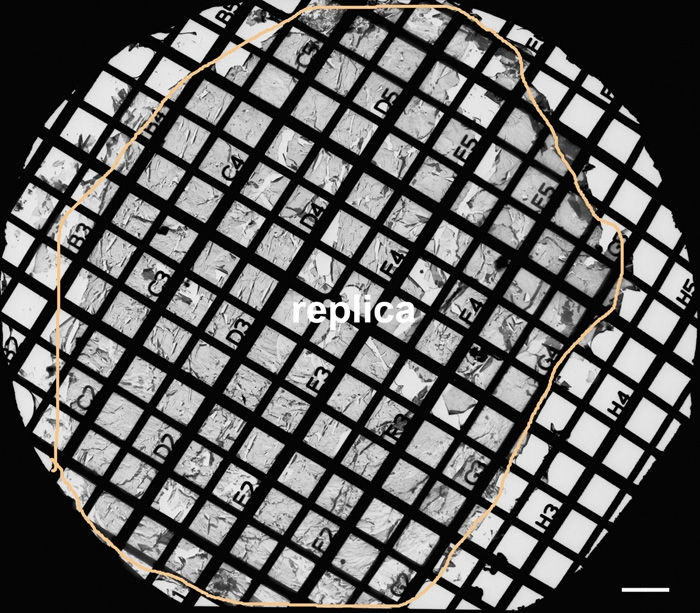
A large intact replica on the Girder finder grid with index number for systematic examinations of fiber gap junctions in the embryonic chicken lens. A low magnification shows an overview of a large intact replica (outlined by circle line) on the Girder finder grid with index number for systematic examinations of fiber gap junctions from outer to inner cortical regions of the embryonic chicken lens at E15. The peripheral area along the circle line represents the superficial surface, and the center represents the nucleus of the lens. This freeze-fracture replica was prepared from a 300 μm lens slice initially cut with a Vibratome for the longitudinal orientation of cortical fiber cells. In this study, initial identifications of various lens regions were made and recorded using the index number (the distance between two parallel grid bars is 100 μm), followed by thorough examinations of the structures of interest in each region. The scale bar is equal to 100 μm.

The major advantage of this technique is that the en face view of antibody labeling can be clearly visualized at the high resolution EM level. The FRIL was conducted according to the procedures of Zampighi et al. [27,28] with modifications. We have used sagittal (longitudinal) lens slices (300 μm thick) prepared from Vibratome to routinely make large freeze-fracture replicas (Figure 1). By using this approach, we are able to conduct systematic examinations of antibody localization in various lens regions at the EM level.

In brief, chicken lenses were lightly fixed in 0.75-1% paraformaldehyde in PBS for 30-45 min at RT, and then cut into 300 mm slices with a Vibratome for making freeze-fracture replicas. Parloidion was used to secure the integrity of the whole piece of a large replica during cleaning and immunogold labeling procedures. Replica was washed with 2.5% sodium dodecyl sulfate, 10 mM Tris-HCl, 30 mM sucrose, pH 8.3 (SDS buffer) until all visible attached tissue debris was removed from the replica. Replica was then rinsed with PBS, blocked with 4% BSA-0.5% teleostean gelatin in PBS for 30 min, incubated with individual Cx45.6 or Cx56 pAb (a kind gift from Dr. Jean Jiang of UT San Antonio) at 1:10 dilution for 1 h at RT. Replica was washed with PBS and incubated with 10 nm Protein A gold (EY Laboratories, San Mateo, CA) at 1:50 dilution for 1 h at RT. After rinse, replica was fixed in 0.5% glutaraldehyde in PBS for 10 min, rinsed in water, collected on a 200-mesh Gilder finder grid, rinsed with 100% amyl acetate for 30 s to remove parloidion, and viewed with JEOL 1200EX TEM.

In addition, we applied our newly developed filipin-FRIL technique to simultaneously visualize cholesterol and connexins labeling in gap junction plaques at the EM level. In brief, the freshly isolated chicken lens was fixed in 1% paraformaldehyde-PBS for 30 min, 300 mm Vibratome sections were cut and lens slices were processed for filipin cytochemistry for 24 h as described above. Freeze-fracture replica was then prepared, washed with SDS buffer and processed for immunogold labeling of Cx56 or Cx45.6 as described above.

In all our immunogold labeling, we used only one side (bottom) of the replica to make contact with the antibody solution as done by Zampighi et al. [27] for the control of non-specific labeling in FRIL. This was achieved by significantly shortening the washing time in SDS solution so that the replica was floating on the surface, instead of sinking entirely into the solution. This was a crucial step to ensure that the replica was properly positioned during the entire following antibody labeling procedures. Also, the top or bottom side of the replica could easily be recognized by the use of parloidion only on the top side of the replica. This surface identification prevented accidentally presenting the wrong (top) side for antibody labeling. We used only the floating replicas for all our immunogold labeling and the labeling time was short, and this eliminated the possibility that the gold labeling particles could be seen from both sides of the replica.

### Quantitative analysis

For determining the amounts of filipin-cholesterol complexes (FCCs) particles (25-35 nm in diameter) on gap junction plaques, intact large freeze-fracture replicas were made using mid-sagittal Vibratome sections (300 μm thick slices) from chicken embryonic lenses (E15-18). The intact whole replicas (about 1,500 μm in diameter) were prepared to include the regions of interest: the outer cortex (0-200 μm from the surface) and the inner cortex (200-400 μm from the surface). Special Gilder finder grids (EM Sciences, Hatfield, PA), which show index numbers, were used for initial identifications of various lens regions followed by systematic examinations. The measurements were made from the peripheral area of the replica, which represents the superficial lens surface, toward the center area which represents the nucleus of the lens. In each replica, the number of FCCs in gap junction plaques was counted manually with a cell counter from the electron micrographs taken at 25,000X. The micrographs of gap junctions were taken randomly from flat cell membranes in the outer and inner cortex from the same replica. Each gap junction area (μm^2^) was measured with the Zeiss AxioVision LE 4.4 on PC (Zeiss Inc., Thornwood, NY). The number of FCCs per μm^2^ GJ area was calculated to determine the subtype of each cholesterol-gap junction measured (i.e., 101-300 and above is considered to be cholesterol-rich; 51-100 is cholesterol-intermediate; and 0-50 is cholesterol-poor and -free). Counting of FCCs was made from 3 replicas prepared from 3 different animals of similar ages. Statistical comparison of the mean percentage of cholesterol-containing gap junctions between cortices was made for each subtype by T-test using the software SPSS 14.0 (SPSS Inc., Chicago, IL). A p<0.05 was considered significant.

## Results

### Formation and maturation of gap junctions in cholesterol-rich cell membranes in lens cortical fibers

**Figure 2:**
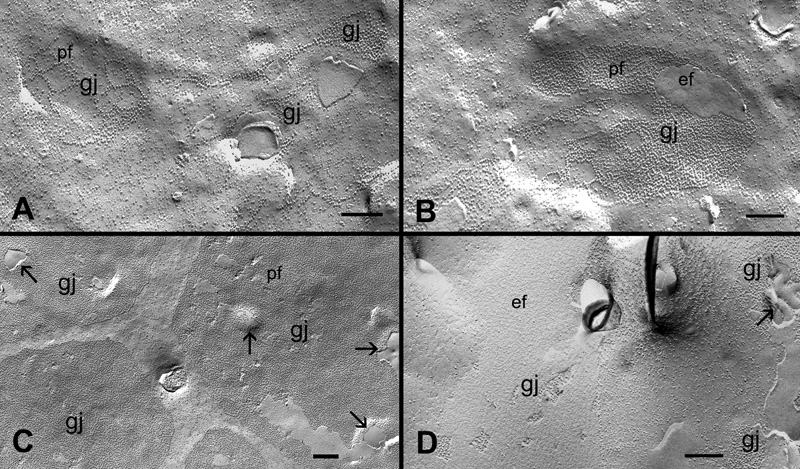
Different structural configurations of gap junctions in various cortical regions of the embryonic chicken lens. Representative micrographs show different structural configurations of gap junctions (gj) found in various cortical regions in the embryonic chicken lens. **A** and **B**: The assembly of forming gap junction plaques in the superficial fibers. **C**: A cluster of well-formed gap junction plaques in the outer cortical fibers. **D**: Several mature gap junction plaques found in the inner cortical fibers. Note the presence of vesicular structures (arrows) associated with gap junctions found in both outer and inner cortical fibers. pf, P-face of the membrane; ef, E-face of the membrane. The scale bars are equal to 200 nm.

With our systematic approach using large intact replicas (Figure 1), we have examined gap junction plaques in the outer (0-200 μm from the capsule surface) and inner (200-400 μm from the surface) cortices in the controls and filipin-treated lens tissues in this study. Figure 2A and Figure 2B show that many irregular shapes and sizes of newly-formed gap junctions can be seen in superficial fiber cell membranes in the outer cortex. Some forming gap junctions exhibit particle free areas within the junctional plaques (Figure 2A,B). A number of well-formed gap junctions as judged by their even distribution of connexons and their regular, smooth border were frequently observed in the outer cortex (Figure 2C). In the inner cortex, some mature gap-junction plaques with a similar loose arrangement of connexons are shown in Figure 2D. In addition, there are distinct vesicular structures specifically associated with gap junction plaques in both outer and inner cortical fibers (arrows in Figure 2C,D). These vesicular structures are devoid of gap junction particles (connexons) and are shown to contain cholesterol (see Results, below for details).

**Figure 3:**
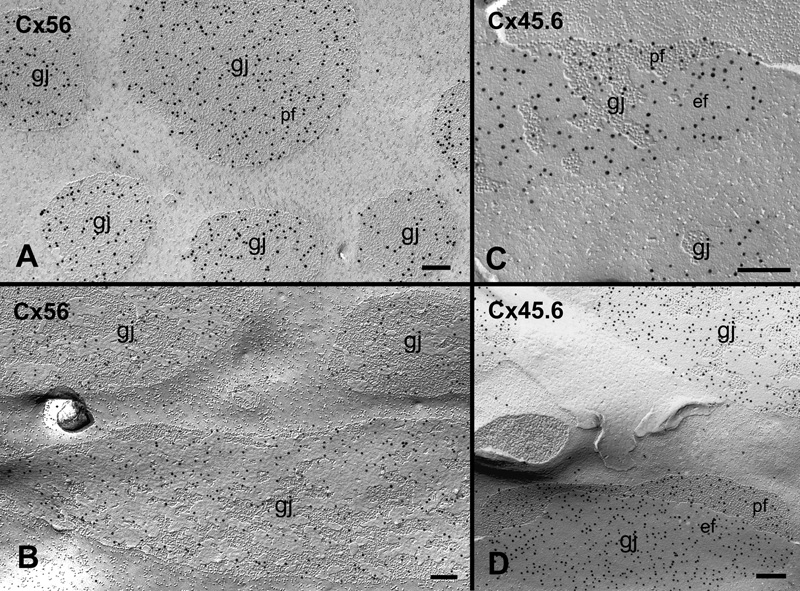
Freeze-fracture replica immunogold labeling of Cx56 and Cx45.6 in gap junctions of the embryonic chicken lens. Freeze-fracture replica immunogold labeling (FRIL) demonstrates that both Cx56 and Cx45.6 polyclonal antibodies are specifically localized in gap junction plaques distributed in the outer cortex (**A** and **C**) and inner cortex (**B** and **D**), respectively. The scale bars are equal to 200 nm.

Freeze-fracture replica immunogold labeling confirms that these gap junction plaques contain both Cx56 and Cx45.6 in chicken lens fibers. Figure 3 shows that Cx56 and Cx45.6 antibodies are specifically localized in gap junction plaques in both outer and inner cortical fibers of embryonic chicken lenses.

### Gap junctions contain different amounts of cholesterol in both outer and inner cortical fiber zones

**Figure 4:**
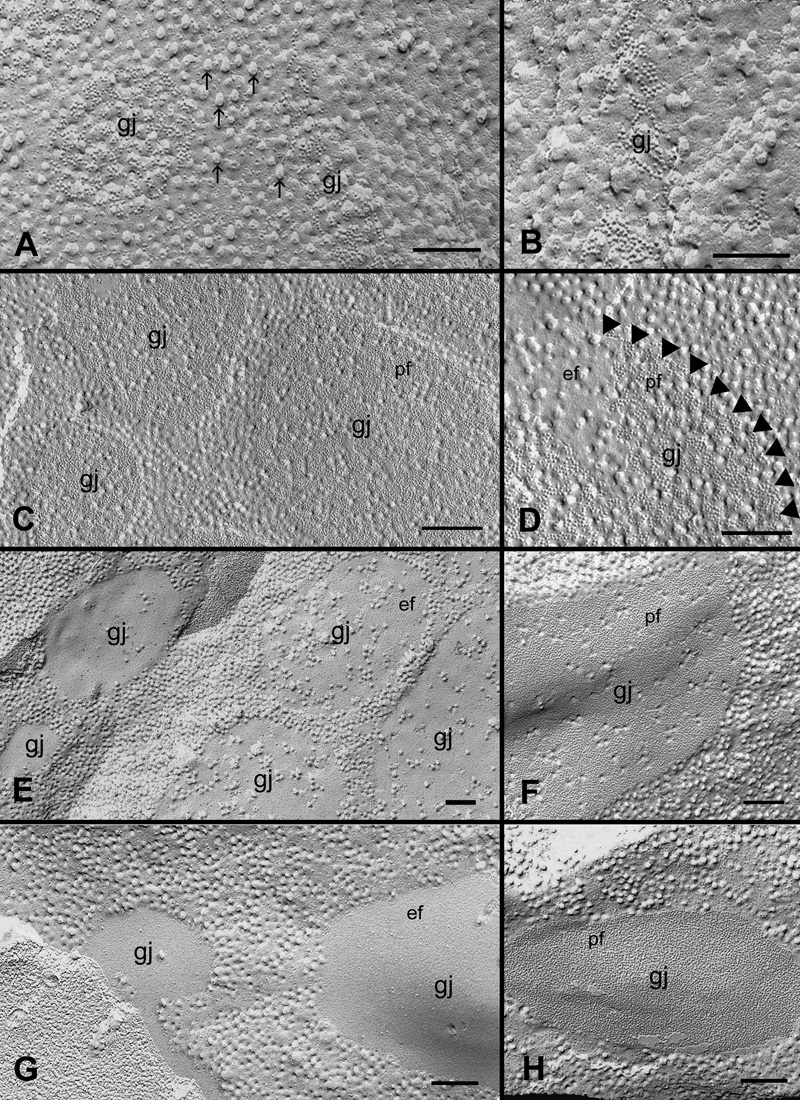
Heterogeneous distribution of cholesterol in gap junction plaques in the outer cortical fiber cells. Gap junctions in the outer cortex (0-200 μm deep) contain different amounts of cholesterol as determined by filipin cytochemistry and freeze-fracture TEM. Newly formed gap junctions (**A** and **B**) and well-formed cholesterol-rich gap junctions (**C** and **D**) contain a large number of filipin-cholesterol complexes (FCCs). Some FCCs (25-35 nm particles) in the P-face of the non-junctional membrane are indicated by the arrows in **A**. At high magnification, the border of such cholesterol-rich gap junction is outlined by arrowheads for a clearer visualization (**D**). Cholesterol-intermediate gap junctions contain considerably less FCCs (**E** and **F**). Cholesterol-poor or -free gap junctions contain only a few or no FCCs in the junctional plaques (**G** and **H**). pf, P-face of the membrane; ef, E-face of the membrane. The scale bars are equal to 200 nm.

**Figure 5:**
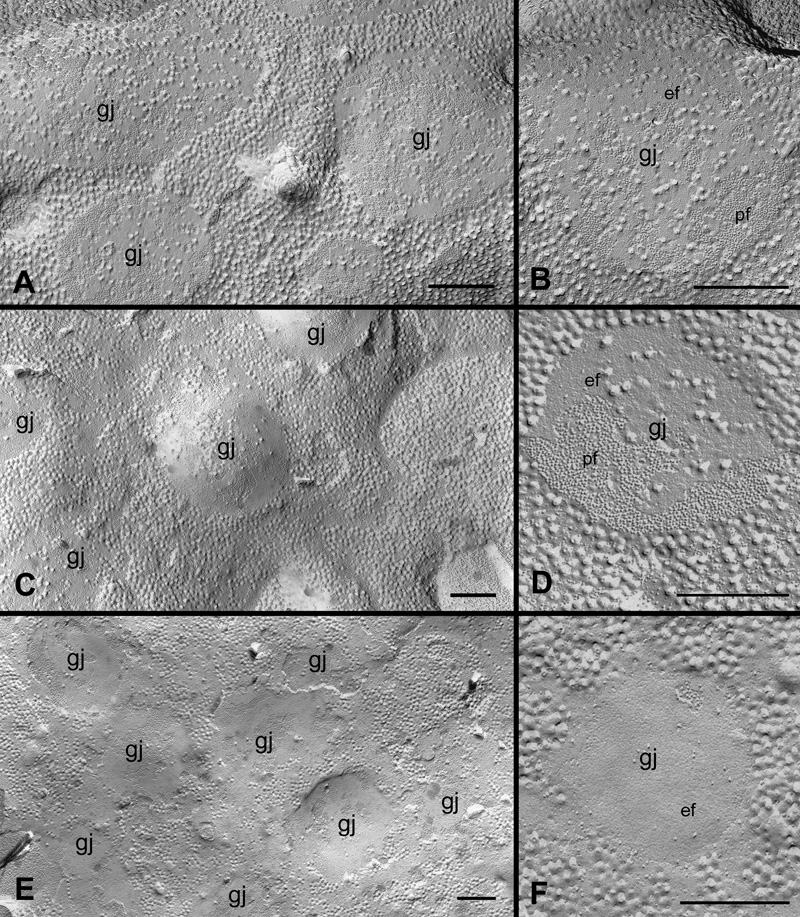
Heterogeneous distribution of cholesterol in gap junction plaques in the inner cortical fiber cells. Gap junctions in the inner cortex (200-400 μm deep) contain different amounts of cholesterol in the embryonic chicken lens. Three subtypes of cholesterol-rich (**A** and **B**), cholesterol-intermediate (**C** and **D**) and cholesterol-free gap junctions (**E** and **F**) were all found in the inner mature fiber cells of the embryonic lens. The scale bars are equal to 400 nm.

Since newly formed gap junctions are assembled in the cholesterol-rich cell membrane, it is expected that these gap junctions would be enriched with cholesterol in the junctional plaques during growth. By using filipin cytochemistry and freeze-fracture TEM, we have consistently observed the presence of filipin-cholesterol complexes (FCC) on gap junctions and cell membranes in various lens regions. The FCCs are discrete particles or pits (25-35 nm in diameter) which can be clearly visualized on the P face and E face of plasma membranes with freeze-fracture TEM. The formation of FCC is due to the polyene antibiotic filipin reacting specifically with membrane cholesterol which produces characteristic membrane lesions seen as the FCC. In superficial fibers, newly formed gap junctions were shown to contain a rich distribution of cholesterol (Figure 4A,B). However, a significant difference in the distribution of cholesterol in the well-formed gap junction plaques was found in both outer and inner cortical fiber cells (Figure 4 and Figure 5). We have classified these gap junctions as cholesterol-rich (101-300 FCCs/μm^2^ GJ area), cholesterol-intermediate (51-100 FCCs/μm^2^ GJ area), and cholesterol-poor-free (0-50 FCCs/μm^2^ GJ area) groups based on their cholesterol contents determined by the number of FCC per μm^2^ gap junction area. Based on our estimations in this study, it is interesting to note that although a large amount of cholesterol are distributed in the gap junctions, the ratio of cholesterol contents between the average cholesterol-rich gap junctions and the adjacent non-junctional membranes is approximately 1:2.

**Figure 6:**
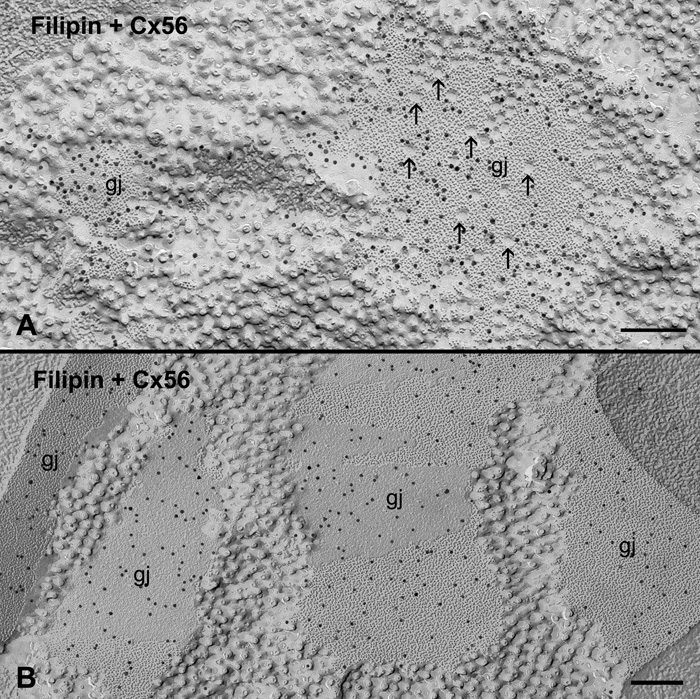
Immunogold labeling of Cx56 in cholesterol-containing gap junctions by innovative technique combining filipin cytochemistry and FRIL. In view of the presence of similar aquaporin junctions in lens fibers, our innovative technique combining filipin cytochemistry and FRIL demonstrates that both cholesterol-intermediate (**A**) and cholesterol-free (**B**) junctions are real gap junctions (gj) because they contain connexin Cx56. Arrows denote some filipin-cholesterol complexes in (**A**). The scale bars are equal to 200 nm.

In view of the presence of similar aquaporin junctions which contain aquaporin 0 in fiber cells as demonstrated by Zampighi et al. [27], our innovative technique combining filipin cytochemistry with FRIL was used to verify that these cholesterol-containing junctions are real gap junctions. Figure 6 shows that the Cx56 antibody was specifically localized in the cholesterol-containing gap junctions (Figure 6A) and the cholesterol-free gap junctions (Figure 6B) in embryonic chicken lens fiber cells.

### Quantitative analysis reveals significant transformation of cholesterol-rich gap junctions into cholesterol-poor and -free gap junctions during fiber cell maturation

The quantitative data obtained from gap junction plaques analyzed from both outer and inner cortices are presented in Table 1. This data was obtained from three replicas prepared from three different chick embryos of similar ages (E15-18). The data is expressed as mean±SD. In each replica, the percentage of a given cholesterol-GJ subtype is determined by the total GJ area of that given subtype divided by the sum of GJ area from the three subtypes. A T-test shows that the decrease on the mean percentage of cholesterol-rich gap junctions from outer cortex (86.6%) to inner cortex (10%) is statistically significant (p<0.001). The increase on the mean percentage of cholesterol-poor-free gap junctions from outer cortex (4.7%) to inner cortex (80.7%) is also statistically significant (p<0.001). A total of 124 and 140 gap junctions were used for quantitative analysis in the outer cortex and inner cortex, respectively. It should be noted that the main objective of this quantitative analysis is to determine the distribution difference for the three subtypes of cholesterol-gap junction plaques in these two cortical regions. The absolute number of cholesterol molecules cannot be pursued by this approach and is not within the scope of this study.

**Table 1:** Distribution of the three subtypes of cholesterol-containing gap junctions in the outer and inner cortical fibers of embryonic chicken lenses.

Outer Cortex (0-200 μm from surface)	Chole-GJ subtypes (FCC/μm^2^GJ area)	Mean GJ area (μm^2^)	Mean FCC per μm^2^ GJ area	Mean percentage of Chole-GJ subtypes
	Chole-poor-free (0-50)	0.9 ±0.6	15 ± 10	4.7 ± 3.2*
	Chole-intermed. (51-100)	2.92 ± 3.63	94 ±5	8.7 ±8.1
	Chole-rich (101-300)	22.32 ±9.81	178 ±6	86.6 ±4.9**
Inner Cortex (200-400 μm from surface)	Chole-GJ subtypes (FCC/μm^2^GJ area)	Mean GJ area (μm^2^)	Mean FCC per μm^2^ GJ area	Mean percentage of Chole-GJ subtypes
	Chole-poor-free (0-50)	16.91 ±8.3	18 ± 10	80.7 ±2.1*
	Chole-intermed. (51-100)	1.89 ±0.9	78 ±7	9.3 ±4.0
	Chole-rich (101-300)	2.05 ± 1.1	139 ± 11	10.0 ±4.0**

The data is expressed as mean±SD. The percentage of a given cholesterol-GJ subtype in each replica (N=3) is determined by the total GJ area of that given subtype divided by the sum of GJ area from the three subtypes. The double asterisk denotes that by T-test the decrease on the mean percentage of cholesterol-rich gap junctions from the outer cortex to inner cortex is statistically significant (p<0.001). The asterisk denotes that the increase on the mean percentage of cholesterol-poor-free gap junctions from the outer cortex to inner cortex is also statistically significant (p<0.001). A total of 124 and 140 gap junctions were used for quantitative analysis in the outer cortex and inner cortex, respectively. FCC, filipin-cholesterol complexes.

### Cholesterol-containing vesicular structures are specifically associated with gap junction plaques in both outer and inner cortical fibers

**Figure 7:**
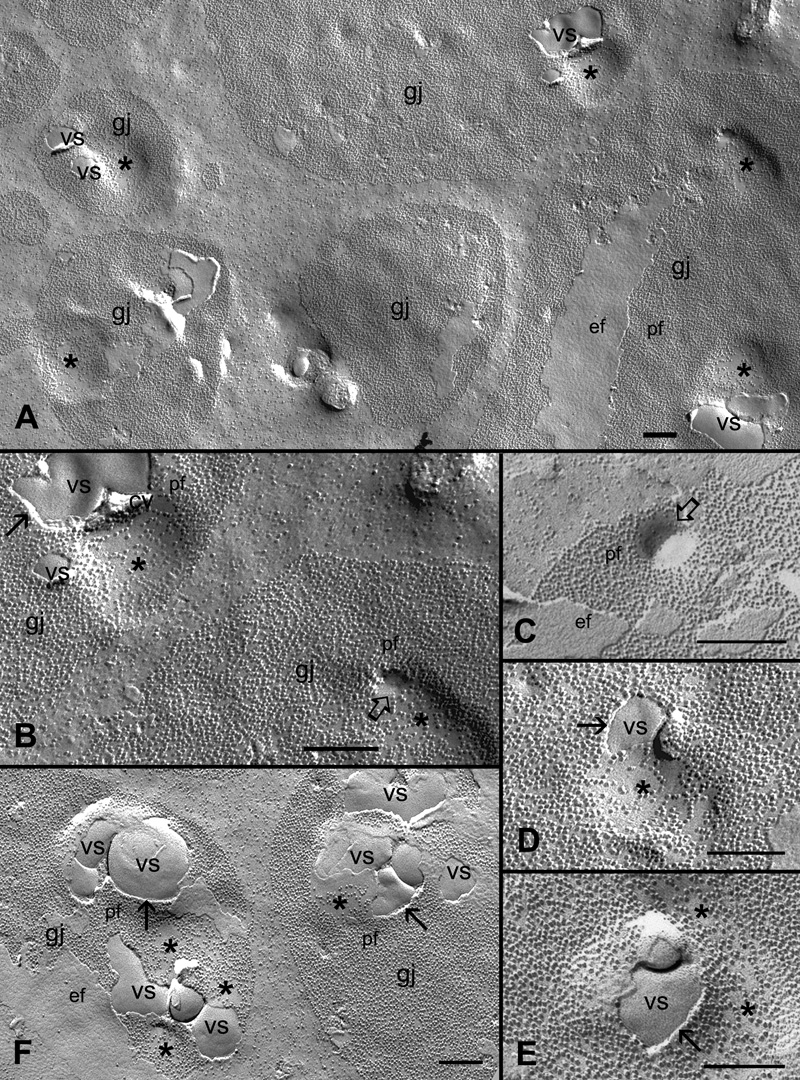
Specific association of unique vesicular structures with gap junction plaques in the outer cortical fibers. **A**: A low magnification shows a number of vesicular structures (vs) specifically associated with gap junction plaques (gj) in the outer cortical fibers. Asterisks denote the presence of small concaved connexon particles-less or -free areas often associated with the vesicular structures. **B** and **C** show the indication of endocytosis (open arrow) of the P-face (pf) of the connexon-free membrane within the gap junction plaques. **D** and **E** exhibit the close association of vesicular structures with the adjacent connexon-less membrane areas (asterisk). **F**: A cluster of vesicular structures with different shapes and sizes are accumulated in two adjacent gap junction plaques. The distinct separation between the P-face of the junction membrane and the vesicular structures is indicated by the arrows. These vesicular structures are shown to be enriched with cholesterol (see Figure 9). The scale bars are equal to 200 nm.

**Figure 8:**
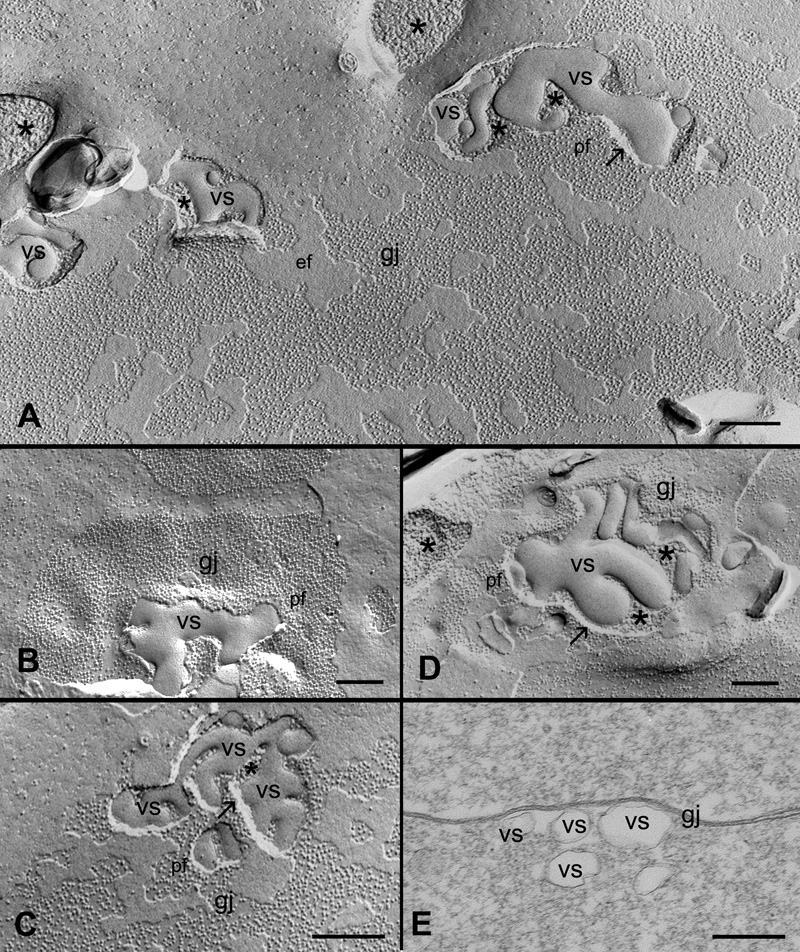
Specific association of unique tubulovesicular structures with gap junction plaques in the inner cortical fibers. A number of vesicular structures with characteristic tubular configurations are observed in gap junction plaques distributed in both outer and inner cortical fibers. They are more often found in the inner cortex (**A**, **C**, and **D**). Asterisks denote the cytoplasm in which the tubulovesicular structures (vs) are located. The distinct separation between the P-face of the junction membrane and the underlying tubulovesicular structures is indicated by the arrows. **E**: Thin-section TEM reveals the presence of a cluster of vesicular structures of a single unit membrane in the cytoplasm closely associated with a fiber gap junction (gj). The scale bars are equal to 200 nm.

**Figure 9:**
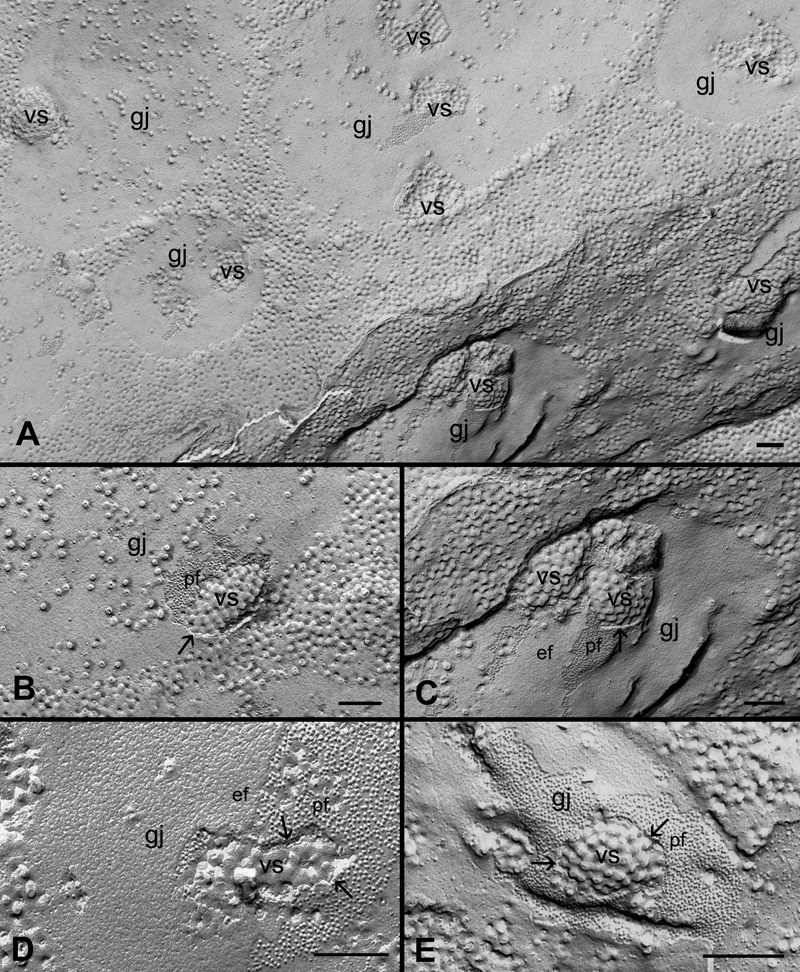
Filipin-treated experiments reveal the presence of discrete filipin-cholesterol particles specifically associated with individual cholesterol vesicles (vs) in the different subtypes of cholesterol-containing gap junctions (gj). **A**: A low magnification shows a number of cholesterol vesicles distributed randomly within the gap junction plaques. **B** through **E** Higher magnifications illustrate the specific accumulation of filipin-cholesterol particles in the vesicular structures (vs) of various forms. The arrows indicate the distinct separation of individual cholesterol vesicles from the gap junction membranes. The scale bars in **A** through **C** are equal to 200 nm and in **D** and **E** are equal to 100 nm.

During the course of our study, a number of vesicular structures with pleiomorphic shapes and various sizes (ranging from 100 nm to >600 nm in diameter) were frequently observed within the gap junction plaques in the control and filipin treatment groups (Figure 7, Figure 8, and Figure 9). These vesicular structures were distributed randomly within the junctional plaques but were often seen near the periphery of the plaques. They exhibited a characteristic smooth surface without gap junction particles (connexons), suggesting that they are lipid in nature. Many were in simple vesicular forms (Figure 7), while others were in more complex tubulovesicular configurations (Figure 8) distributed in both outer and inner cortical fibers. The chance for visualization of these structures was facilitated by the exposure of the fractured P face of the membrane (indicated by black arrows in Figure 7B,D-F) of gap junction plaques, so that the underlying particle-free vesicular structures in the cytoplasm were exposed. Thus, visualization of the simple and complex forms of the vesicular structures appeared to be determined by different degrees of exposure by the freeze-fracturing process. In this case, for example, the exposure of simple vesicular forms would be more frequent for the smooth membrane surface in the outer cortex (Figure 7), while the exposure of the complex tubulovesicular configurations would be more common for the contoured membrane in the inner cortex (Figure 8). In addition, many small concave areas in the junctional membrane which contain a much reduced number of connexons (indicated by asterisks in Figure 7) are found closely associated with the vesicular structures in the gap junction plaques. We hypothesize that these connexon-less areas might be the initial spots for the formation of vesicular structures through endocytosis. We have indeed observed such an early stage of endocytosis which is exemplified by the internalization of a small area of the particle-free gap junction membrane into the underlying cytoplasm (indicated by open arrows in Figure 7B,C). The subsequent fusion (closure) of the endocytotic membrane would result in the formation of vesicular structures in the cytoplasm directly under the gap junction membrane as those observed by the thin-section TEM (Figure 8E).

Since all vesicular structures examined are devoid of gap junction particles, they are most likely composed of lipid, possibly cholesterol. We have indeed observed that all vesicular structures found in the three subtypes of cholesterol-containing gap junctions are enriched with cholesterol (Figure 9) as determined by filipin cytochemistry and freeze-fracture TEM.

## Discussion

This study presents for the first time that gap junctions in the fiber cells of embryonic chicken lenses contain different amounts of cholesterol as determined by filipin cytochemistry and freeze-fracture TEM. For the purpose of determining their heterogeneous distributions in different cortical regions of the lens, we have classified these cholesterol-containing gap junctions as three subtypes based on the number of filipin-cholesterol complexes (FCCs) per unit area (i.e., μm^2^) of gap junction plaques. Namely, cholesterol-poor and -free subtypes contain 0-50 FCCs/μm^2^ Gj area, cholesterol-intermediate subtypes contain 51-100 FCCs/μm^2^ Gj area, and cholesterol-rich subtypes contain 101-300 FCCs/μm^2^ Gj area. By using the same technique, the presence of cholesterol-free gap junctions has previously been found in the aging human lens [25]. In addition, in view of the presence of the similar aquaporin junctions which contain aquaporin 0 [27] in lens fiber cells, the nature of these cholesterol-containing junctions has indeed been confirmed as real gap junctions by the labeling of a specific chicken lens Cx56 antibody using our innovative technique combining filipin cytochemistry with freeze-fracture replica immunogold labeling (Figure 6).

By using our systematic examinations, we have revealed that these three subtypes of cholesterol-containing gap junctions are distributed concurrently in various cortical regions but with different frequencies. Quantitative analysis shows that cholesterol-rich gap junctions are the major populations (about 86%) in the outer young cortical fibers, while the cholesterol-poor and -free gap junctions (about 81%) are primarily distributed in the inner mature cortical fibers (Table 1). Quantitative analysis further reveals that the decrease on the mean percentage of cholesterol-rich gap junctions from outer cortex (86.6%) to inner cortex (10%) is statistically significant (p<0.001). The increase on the mean percentage of cholesterol-poor-free gap junctions from outer cortex (4.7%) to inner cortex (80.7%) is also statistically significant (p<0.001). This result suggests that a majority of cholesterol-rich gap junctions in the outer cortex have been transformed into cholesterol-free ones in the inner cortex during fiber cell differentiation and maturation.

It is interesting to note that while the amount of cholesterol in the gap junction plaques decreases as the fiber cells mature and age, the cholesterol content in the adjacent non-junctional membranes is not reduced in the inner and deep cortex as judged by our filipin-freeze-fracture TEM analysis (Figure 5). Also, because the number and size of gap junction plaques are significantly reduced or eliminated in the deep cortex and the nucleus due to the age-associated degradation [29-34], the cholesterol contents in the total fiber cell membranes would be higher in the deep cortex and the nucleus than that in the more superficial inner cortex because cholesterol-free gap junctions are more abundantly distributed in the inner cortex as reported in this study. This regional disparity in the distributions of cholesterol-containing gap junctions may explain the earlier biochemical data showing that the cholesterol contents of fiber cell membranes are gradually increased in the deep cortex and the nucleus in several species studied [5,8,35].

The finding that gap junctions in the superficial fibers are enriched with cholesterol is not surprising since new gap junctions are all assembled in the cholesterol-rich fiber cell membrane [1,4,5,8], and, as a result, all newly-formed gap junctions are expected to be enriched with cholesterol in the superficial cortical fibers. However, the subsequent increase of the cholesterol-poor and -free gap junction population in the inner cortical fibers during fiber cell maturation is interesting. This suggests that the cholesterol-rich gap junctions must undergo a cholesterol removal process in order to become the cholesterol-free ones. The concomitant presence of three subtypes of cholesterol-containing gap junctions in the outer superficial cortical fibers suggests that some cholesterol-rich gap junctions have already begun the cholesterol removal process in young cortical fibers. Since fiber gap junctions have a relatively longer half-life and do not undergo fast turnover like those in other cell types [13,36,37], it is reasonable to expect that with the continual removal of cholesterol from gap junction plaques as fiber cells mature, a majority of cholesterol-rich gap junctions would be transformed into the cholesterol-free ones in the inner cortex. Our quantitative analysis has indeed supported this assertion (Table 1).

The removal of cholesterol molecules from the gap junction plaques is not expected to be carried out through the passive lateral diffusion to the surrounding non-junctional membranes, because the lateral diffusion process would be against the higher gradient of cholesterol in the surrounding non-junctional membranes. In our estimations in this study, the ratio of cholesterol contents between the average cholesterol-rich gap junctions and the adjacent non-junctional membranes is approximately 1:2. Thus, it is expected that removal of cholesterol would be conducted through the similar endocytosis mechanism for gap junction connexins [14-16]. The endocytosis of gap junction connexins is initiated by invagination and followed by internalization of double unit membrane of gap junction fragments (i.e, annular junctions) into the cytoplasm [15,16,38-40]. The subsequent degradation and recycling of connexins is further carried out by proteasome and lysosome [15,16,41]. In addition, several previous studies conducted in the live cells have shown that older connexins in pleiomorphic vesicles of widely varying sizes are removed by endocytosis from the center of junctional plaques into the cytoplasm [14,17] while the newly synthesized connexins in 100-150 nm vesicles are incorporated at the periphery of existing gap junctions [14]. Windoffer et al. [17] were the first to show in their live cell study that long tubulovesicular structures of gap junction fragments were dynamically invaginated and pinched off into the cytoplasm during endocytosis.

Because gap junctions in other cell types do not contain large amounts of cholesterol, it is unique in the lens that endocytosis of lens fiber gap junctions would involve both cholesterol and connexins. Surprisingly, in the present study we have discovered a distinct removal process for cholesterol alone from gap junction plaques in the lens (Figure 7, Figure 8, and Figure 9). This removal process appears to be similar to the endocytosis of connexins described in other cell types; the endocytosed cholesterol-rich vesicles are also in pleiomorphic shape and widely varying in sizes. The existence of internalized cholesterol-rich vesicles is evidenced by the absence of gap junction connexon particles and the presence of filipin-cholesterol complexes as examined with freeze-fracture TEM and filipin cytochemistry (Figure 7, Figure 8, and Figure 9). The formation of cholesterol-rich vesicles appears to be initiated by the dispersal of connexon particles to create some small connexon-less or -free membrane areas in the junctional plaques before (Figure 7A-F) and during (Figure 7B,C) endocytosis. In addition, it is worthy of note that while the endocytosis of connexins occurs only from the center of junctional plaques [14], cholesterol-rich vesicles are internalized in various locations and are often near the periphery of junctional plaques (Figure 7, Figure 8, and Figure 9). It is reasonable to predict that the continual removal of cholesterol-rich vesicles would eventually convert the cholesterol-rich gap junctions into the cholesterol-free ones during fiber cell maturation. It should be noted that although the typical endocytosis of gap junction connexins has not yet been observed in the present study, we do not rule out the possibility that connexins in fiber cells may undergo different forms of degradation during fiber cell maturation and aging.

How the cholesterol molecules are drawn together to small specific areas within the gap junctional domain for endocytosis is another interesting question. This may involve active trafficking of cholesterol by certain cholesterol-binding proteins such as caveolin-1 [42-44]. Caveolin-1 is present in the lens fiber cells and interacts with gap junction connexins [45-47]. The specific localization of caveolin-1 in the gap junction plaques in the chicken lens fiber cells has recently been demonstrated by freeze-fracture replica immunogold labeling in our laboratory [48]. Since caveolin-1 is a cholesterol binding protein and involved in cholesterol transport [42-44], it is highly possible that caveolin-1 binds and transports cholesterol to specific areas within the gap junction domain for subsequent endocytosis of cholesterol vesicles.

It is not known why the cholesterol in the cholesterol-rich gap junctions needs to be removed to become the cholesterol-free ones during fiber cell maturation. It is speculated that the transformation of gap junction structural configurations from outer to inner cortical fibers in relation to cholesterol contents may be a necessary change for gap junctions to adapt to the more acidic environment in the deep regions of the lens. It is known that gap junctions in the superficial differentiating fiber zone are pH sensitive while those in the mature fiber zone are pH insensitive as demonstrated in vivo in several species by Mathias et al. [18,49,50] and Lin et al. [51]. It has been suggested that because deep in the lens, the fiber cell cytoplasm is significantly more acidic (pH 6.81), the loss of gap junction sensitivity to pH may be crucial to lens survival [49,50]. It is also possible that removal of cholesterol from gap junctions would make the junction plaques more fluid, and this membrane property would be more suitable for close interactions among connexons presumably required for their unique gating activities in the mature fiber cells. However, although it is not known the effect of cholesterol itself on the gap junction physiology, the rich and poor distributions of cholesterol would certainly affect the connexon packing and the structural configurations of gap junctions in the lens. This possibility is currently under systematic investigations in the adult chicken lens.
